# Development of a diagnostic rule for identifying radiographic osteoarthritis in people with first metatarsophalangeal joint pain

**DOI:** 10.1186/1757-1146-4-S1-O53

**Published:** 2011-05-20

**Authors:** Gerard V Zammit, Shannon E Munteanu, Hylton B Menz

**Affiliations:** 1 Musculoskeletal Research Centre, Faculty of Health Sciences, La Trobe University, Bundoora, Victoria 3086, Australia; 2 Department of Podiatry, Faculty of Health Sciences, La Trobe University, Bundoora, Victoria 3086, Australia

## Background

Diagnosis of osteoarthritis (OA) affecting the first metatarsophalangeal joint (MTPJ) is typically made on the basis of clinical observations such as restricted dorsiflexion range of motion of the first MTPJ, the presence of a dorsal exostosis and secondary complications such as hyperextension of the first interphalangeal joint. However, the ability of these observations to accurately identify the presence of first MTPJ OA has not been examined. Therefore, the aim of this study was to develop a diagnostic rule for the identification of radiographic OA of the first MTPJ in people with first MTPJ pain.

## Methods

Symptoms and clinical observations were documented in 181 people with first MTPJ pain, and the presence of OA was confirmed using plain film radiography. Diagnostic test statistics were calculated to assess the ability of symptoms and clinical observations to identify radiographic OA. Multivariate logistic regression was used to develop two diagnostic models: a statistically optimal model and a simplified clinical model.

## Results

Multivariate logistic regression identified pain duration greater than 25 months, the presence of a dorsal exostosis, hard-end feel, crepitus and less than 64 degrees of first MTPJ dorsiflexion to be significantly associated with radiographic OA. The statistically optimal model and clinical model performed similarly, with the areas under the receiver operating characteristics curves being 0.87 (95% confidence interval [CI] 0.81 to 0.93) and 0.87 (95% CI 0.80 to 0.93), respectively, and the percentage of cases correctly classified being 86.2 and 85.6, respectively. A cut-off score of ≥ 3 using the clinical model resulted in a sensitivity of 88%, specificity of 71%, accuracy of 84%, positive likelihood ratio of 3.07 and negative likelihood ratio of 0.17.

## Conclusions

In people with first MTPJ pain, a model consisting of five clinical observations can accurately identify the presence or absence of radiographic OA. The application of this diagnostic rule may assist clinical decision making and potentially reduce the need for referral for radiographs.

